# Host-Specific Diversity of Culturable Bacteria in the Gut Systems of Fungus-Growing Termites and Their Potential Functions towards Lignocellulose Bioconversion

**DOI:** 10.3390/insects14040403

**Published:** 2023-04-21

**Authors:** Rongrong Xie, Chenchen Dong, Shengjie Wang, Blessing Danso, Mudasir A. Dar, Radhakrishna S. Pandit, Kiran D. Pawar, Alei Geng, Daochen Zhu, Xia Li, Qing Xu, Jianzhong Sun

**Affiliations:** 1 Biofuels Institute, School of the Environment and Safety Engineering, Jiangsu University, Zhenjiang 212013, China; 2 Department of Zoology, Savitribai Phule Pune University, Pune 411007, India; 3 School of Nanoscience and Biotechnology, Shivaji University, Kolhapur 416004, India

**Keywords:** fungus-growing termites, *Ancistrotermes pakistanicus*, gut systems, bacterial diversity, symbionts, lignocellulose digestion, culturable bacteria

## Abstract

**Simple Summary:**

Termites represent a unique and highly efficient system for lignocellulose bioconversion. The highly efficient lignocellulolytic systems are achieved through contributions from gut bacterial symbionts. Despite extensive research, the complete picture of bacterial diversity and their associated functions towards lignocellulose digestion by fungus-growing termite species is still lacking. In line with this objective, the present study revealed a diversity profile of cellulolytic and hemicellulolytic bacterial symbionts in the gut systems of some representative fungus-growing termites. The potential functions of the bacteria towards lignocellulose digestion, particularly cellulose and hemicellulose hydrolysis, were further identified and analyzed. The present investigation provided a unique profile of lignocellulose degrading symbiotic bacteria from the termites in general and fungus-growing species in particular.

**Abstract:**

Fungus-growing termites are eusocial insects that represent one of the most efficient and unique systems for lignocellulose bioconversion, evolved from a sophisticated symbiosis with lignocellulolytic fungi and gut bacterial communities. Despite a plethora of information generated during the last century, some essential information on gut bacterial profiles and their unique contributions to wood digestion in some fungus-growing termites is still inadequate. Hence, using the culture-dependent approach, the present study aims to assess and compare the diversity of lignocellulose-degrading bacterial symbionts within the gut systems of three fungus-growing termites: *Ancistrotermes pakistanicus, Odontotermes longignathus*, and *Macrotermes* sp. A total of 32 bacterial species, belonging to 18 genera and 10 different families, were successfully isolated and identified from three fungus-growing termites using Avicel or xylan as the sole source of carbon. *Enterobacteriaceae* was the most dominant family represented by 68.1% of the total bacteria, followed by Yersiniaceae (10.6%) and Moraxellaceae (9%). Interestingly, five bacterial genera such as *Enterobacter*, *Citrobacter*, *Acinetobacter, Trabulsiella*, and *Kluyvera* were common among the tested termites, while the other bacteria demonstrated a termite-specific distribution. Further, the lignocellulolytic potential of selected bacterial strains was tested on agricultural waste to evaluate their capability for lignocellulose bioconversion. The highest substrate degradation was achieved with *E. chengduensis* MA11 which degraded 45.52% of rice straw. All of the potential strains showed endoglucanase, exoglucanase, and xylanase activities depicting a symbiotic role towards the lignocellulose digestion within the termite gut. The above results indicated that fungus-growing termites harbor a diverse array of bacterial symbionts that differ from species to species, which may play an inevitable role to enhance the degradation efficacy in lignocellulose decomposition. The present study further elaborates our knowledge about the termite-bacteria symbiosis for lignocellulose bioconversion which could be helpful to design a future biorefinery.

## 1. Introduction

Termites are small and social insects that represent unique models for lignocellulose digestion which can effectively utilize recalcitrant lignocelluloses as their main food source. Termites are found in tropical as well as subtropical regions [1]. To date, more than 3000 species of termites have been described with a few of them acting as pests of wood and crop plants [2,3]. Based on their feeding habits, termites are usually categorized into four groups: wood feeders, soil feeders, grass feeders, and fungus-growing termites [4]. Among termites, fungus-growing species are predominant decomposers that can efficiently digest plant matter, including grasses, leaves, forest litter, and dung [5]. Fungus-growing termites are affiliated with the subfamily *Macrotermitinae*. The highest number of fungus-growing termite species occurs in the forests of Africa while only four genera are represented in Asia. These termites share an external association with Basidiomycete fungus, *Termitomyces* (e.g., members of *Macrotermitinae*), apart from the endosymbiotic prokaryotes [1,6] for lignocellulose digestion. They can degrade over 90% of the lignocellulose in some regions within only 30 min [7,8], therefore demonstrating their prominent impact on overall carbon recycling [5,9]. They are also known as ecosystem engineers due to their role in carbon turnover, enhanced soil fertility, and crop yields [10,11,12,13,14]. Termites achieve this high efficiency of lignocellulose utilization through symbiosis with microbes that shelter in their gut systems [15]. The gut system of termites is considered the world’s smallest, yet most efficient natural bioreactor that can produce up to two liters of hydrogen from a single piece of paper via digestion. The gut system of termites is a very complex microhabitat with unique biotic and abiotic features [16]. These digestomes possess unique ecological niches to shelter a diverse array of microbiota that attribute a variety of functions to the host. Termites rely on the gut microbial symbionts that attribute their host with an arsenal of enzymes [17]. The gut symbionts augment the host to degrade recalcitrant diets such as lignocellulose [5,18] into short-chain fatty acids and methane. Short-chain fatty acids and methane are commercial molecules; therefore, harnessing and understanding the mechanism of lignocellulose bioconversion within the termite gut is necessary for future biorefinery.

The mechanism of lignocellulose digestion within the gut of fungus-growing termites is still unknown. Among higher termites, the digestion of lignocellulose is more complex [19] in fungus-growing species due to their external association with the basidiomycete fungus, *Termitomyces* sp. Although *Termitomyces* sp. is known to facilitate partial digestion of lignocellulose, the impact of this external association on gut bacteria in fungus-growing termites has remained unclear. Moreover, the comprehensive details of the bacterial diversity and the enzymes involved in lignocellulose digestion within the gut of fungus-growing termites are still lacking. Despite adapting the trait of fungi culture, the involvement of the bacterial community in fungus-growing termites may still be important for the highly efficient digestion of lignocellulose. The presence of fungal symbiosis has caused functional division of labor with complementary functions for *Termitomyces* and the gut bacterial community [19,20]. However, the comprehensive details elucidating the functions of bacterial symbionts within the gut system of fungus-growing termites towards lignocellulose digestion are still elusive. 

Over the course of evolution, termites have evolved mutual relationships with microbes, particularly bacteria [3]. The bacterial symbionts act as “microbial brokers” that enable the hosts to overcome the activities that are either low or absent in the host [21,22], thereby providing additional capabilities to the termites [23]. During the last three decades, a plethora of information has been generated related to the presence and functions of bacteria in termite gut systems through high throughput sequencing techniques involving omics [24]. However, the major drawback of these omics methods is the lacunae in terms of details about the contribution of individual bacterial strains. Moreover, the putative functions explained by omics strategies may not always be accurately associated with the particular strains of bacteria. Therefore, a comprehensive understanding of the bacterial diversity within termite guts would highlight the vital features of the bacteria towards their symbiotic mechanisms. Furthermore, understanding the functions of the individual bacteria within the guts of termites might be useful from the basic biology and biotechnology viewpoints. Despite numerous attempts to explore the diversity and functions of gut symbionts, the fundamental knowledge concerning the mechanism of symbiotic lignocellulose digestion in termites is still elusive. Moreover, based on the evolutionary differences and acquisition of the fungal symbiosis [25], but eliminating flagellates as found in lower termites [26], we hypothesized that fungus-growing termites must harbor a unique set of core bacteria that might be involved in biomass degradation. Among fungus-growing termites, *A. pakistanicus* is a facultative inquiline by its lifestyle, occurring in southern China, while *Odontotermes* are important decomposers and crop pests found throughout southern Asia [27]. *Macrotermes* include the largest sized termites found across tropical regions. Although these termites belong to the same group, they differ in many aspects such as habitat, body size, phylogeny, etc., which also signpost that the bacterial composition among these termites might show variations. Moreover, the gut microbiota is known to significantly affect the host’s biology [28,29]. The long-term vertical transmission of the bacterial symbionts in termites is evident from their highly sophisticated social structure and complex gut microbiota, which allow co-evolution of the bacterial symbionts with the host [30,31]. Despite the fact that termites receive the majority of their gut microbiota through the process of proctodeal trophallaxis [32], there is still a debate on how microbial communities are assembled during termite evolution. Therefore, to elaborate the current state of knowledge, the present study assessed the diversity of the bacterial symbionts in three fungus-growing termite species: *A. pakistanicus*, *O. longignathus*, and *Macrotermes* sp. In addition, the potential functions towards digestion of the lignocellulose by bacterial symbionts are also elaborated to understand the mechanism of termite–bacterial symbiosis. Finally, some potential bacterial symbionts were selected and evaluated on corn stover and rice straw under controlled conditions to monitor the production of different lignocellulases (particularly endoglucanase, exoglucanase, and xylanases), substrate degradation, and growth patterns that might be helpful for the design of a future refinery based on termite gut mimetics. 

## 2. Materials and Methods

### 2.1. Materials

#### 2.1.1. Lignocellulosic Substrates

The lignocellulosic raw materials used in this study included agricultural wastes, such as rice straw and corn stover, which are generally incinerated after harvesting the crop. These substrates were collected from the local fields, washed with deionized water to remove impurities, and then air-dried. The air-dried lignocellulosic materials were subsequently grinded in a food processor and sieved through a 2.0 mm strainer to obtain the uniform-sized particles. These carbon sources were either used immediately for hydrolysis experiments or stored in air-tight containers at 4 °C until use.

#### 2.1.2. Growth Media and Reagents

The commercial cellulosic materials carboxymethyl cellulose (CMC), Avicel, and hemicellulose representing birchwood xylan were purchased from Sigma Aldrich, (St. Louis, MO, USA). The media used for the isolation, screening, and culturing of bacteria were basic salt media (BSM) that contained CaCl_2_·2H_2_O, 0.5 g; K_2_HPO_4_, 1.0 g; MgSO_4_·7H_2_O, 0.5 g; FeSO_4_·7H_2_O, 0.02 g; and NaNO_3_, 0.5 g per liter of solution. Each time, the media was prepared fresh and autoclaved at 121 °C for 15–20 min prior to use. All other chemicals used in the study were of the highest purity and molecular or analytical grade unless otherwise mentioned. 

### 2.2. Methods

#### 2.2.1. Termite Gut Collection and Sample Preparations

The fungus-growing species of termites undertaken for the present study viz, *A. pakistanicus*, *O. longignathus*, and *Macrotermes* sp. were collected from the Xishuangbanna tropical rain forest, Yunnan province, China during the summer season. The sampling of the termites was conducted three times during the summer season for each species. Immediately after collection and transportation, the termites were identified through the molecular phylogenetic approach of DNA barcoding. To accomplish this, the gene encoding cytochrome oxidase II was sequenced by using the forward COIIf: 5′-CAGATAAGTGCATTGGATTT-3′ and reverse primers COIIr: 5′-GTTTAAGAGACCAGTACTTG- 3′. The COII gene sequenced data of the termites were submitted to Genbank to obtain the accession numbers (*A. pakistanicus*: OQ716402; *O. longignathus:* OQ713168; *Macrotermes* sp.: OQ713177). Phylogenetic trees were reconstructed for the sequenced termites using the MEGA 7 package alongside the neighbor-joining method with a Kimura-2 model. Thereafter, the collected termites were dissected under sterile conditions to reveal the gut systems. Since only the worker caste of the termites is responsible for feeding and maintaining the colony, in the present study 15 worker caste individuals of each species were taken into consideration for experimentation. Prior to dissection, the worker termites were surface sterilized with 70% ethanol for 60 s followed by a brief wash of sterile double distilled water. The surface sterilized individuals were dissected in a laminar airflow under aseptic conditions using sterilized instruments. The gut systems (15 of each species) were extracted and thoroughly homogenized in 200 μL of a saline solution (0.85%) using micro-pestles followed by the addition of 800 μL of a phosphate buffer saline (PBS) solution to make the final volume 1 mL. 

#### 2.2.2. Growth and Isolation of the Bacteria from Termite Gut Systems

The gut homogenates containing microbes were then vortexed, and 100 µL of each sample was spread on the BSM media plates containing either Avicel or xylan as sole sources of carbon. The BSM media containing different carbon sources were supplemented with trace elements (per liter of distilled water: FeCl_2_·4H_2_O, 1.5 g; CoCl_2_·6H_2_O, 190 mg; MnCl_2_·4H_2_O, 100 mg; ZnCl_2_, 70 mg; H_3_BO_3_, 62 mg; Na_2_MoO_4_.2H_2_O, 36 mg; NiCl_2_·H_2_O, 24 mg; CaCl_2_·H_2_O, 2 mg; 25% HCl, 10 mL ) and vitamin solutions (4-aminobenzoic acid, 40 mg; biotin, 10 mg; nicotinic acid, 100 mg; calcium-pantothenate, 50 mg) to allow the adaptation and initial growth of the bacteria [33,34]. After spreading, the plates were incubated at 30 °C for 3 days. Subsequently, the plates were observed to isolate the unique and distinct colonies that can grow and digest the substrates. The isolated bacteria were purified through repeated sub-culturing and streaking on BSM-Avicel and -xylan plates. The isolated bacteria after purification were codified, identified, and characterized further for lignocellulose biodegradation. Moreover, a stock of the bacteria was preserved at −80 °C as 20% glycerol stock for further use. 

#### 2.2.3. DNA Extraction, PCR Amplification, and Sequencing

To reveal the identity of the isolates, each purified bacterium was grown overnight in Luria Bertani broth at 30 °C. The genomic DNA was extracted using a bacterial gDNA extraction kit^®^ (TakaRa, Beijing, China) and checked on 0.8% agarose gels. The 16S rRNA gene of individual isolates was amplified by running a PCR reaction in a S1000TM Thermal Cycler (BioRad, Hercules, CA, USA). For PCR amplification of the 16S rDNA gene, the bacteria-specific primer pairs such as 27F (5′-AGAGTTTGATCMTGGCTCAG-3′) and 1492R (5′-124 TACGGYTACCTTGTTACGACTT-3′) [35] were used. The 50 μL PCR reaction mixture contained 2 μL (10 ng/µL) of template DNA, 5 μL of Taq buffer, 4 μL of dNTPs (2.5 mmol/L each), 1 μL of each primer (10 pmol), 0.4 μL of Taq DNA polymerase (2 U), and 36.6 μL of RNase-free pure water. The thermal cycling conditions consisted of initial denaturation at 95 °C for 4 min, 30 amplification cycles of primary denaturation at 95 °C for 30 s, annealing at 54 °C for 45 s, extension at 72 °C for 2 min, and a final extension step at 72 °C for 10 min. The PCR reactions were run in triplicates and all amplicons were resolved for size and purity on 1.2% agarose gels by staining with 4S Plus staining solution (Sangon Biotech). The successful PCR amplicons were subsequently sent for sequencing to Sangon Biotech, Shanghai, China. 

#### 2.2.4. Identification and Phylogenetic Analysis of Isolated Bacteria

After successful amplification and sequencing, the raw sequences of the 16S rDNA genes of isolated bacteria obtained were processed for base calling and contig preparation in ChromasPro software (http://www.technelysium.com.au/ChromasPro.html; accessed on 27 December 2022). The chimera-free 16S rRNA sequences representing individual isolates were subjected to BLASTn analysis in the NCBI database and EzBioCloud (https://www.ezbiocloud.net/identify; accessed on 27 December 2022) to obtain the same gene sequences of closely related species. The 16S rDNA gene sequences of the NCBI hits and individual isolates were analyzed for multiple sequence alignment using the Clustal W program. After alignment, the phylogenetic trees were reconstructed in MEGA X software [36] using the neighbor-joining (NJ) method maintaining 500 bootstrap values [37] and then annotated online in the Interactive Tree of Life V5 (iTOL, https://itol.embl.de/) [38]. The taxonomic annotation of the bacteria was determined based on reconstructed phylogenetic trees and then confirmed from the EzBioCloud databases (https://www.ezbiocloud.net/identify; accessed on 27 December 2022) as well as the ribosome database project (RDP).

#### 2.2.5. Deposition of DNA Sequences

The obtained 16S rDNA sequences were submitted to GenBank (http://www.ncbi.nlm.nih.gov) to acquire the accession numbers. The 16S rDNA sequences of the bacterial isolates can be retrieved from the NCBI database under accession numbers ranging from MW947072 to MW947137. 

#### 2.2.6. Screening of the Bacteria for Cellulase and Xylanase Production

After purifying each of the isolates, the primary screening of the bacteria was tested for avicellase (exoglucanase) and xylanase activities. To perform screening, the purified isolates were inoculated to BSM media containing 1% of either Avicel or xylan as the only source of carbon. The culture flasks were incubated at 30 °C for 3 days in a rotary shaker agitating at 180 rpm. After 3 days of culturing, small aliquots of 2 mL from each flask were sampled and centrifuged at 10,000× *g* for 15 min maintaining a temperature of 4 °C. The crude supernatants obtained were treated as enzyme extracts and tested for enzymatic activities. The enzyme assays were performed as described previously [39] with few modifications. The 20 µL of the enzyme extract was briefly mixed with 80 µL of the substrates (1% Avicel or xylan) in 0.05 M sodium phosphate buffer saline (PBS, pH, 6.0). Then, the reactants were incubated at 40 °C for 60 min and 30 min, respectively, for avicellase and xylanase activities. After incubation, the reactions were stopped by adding 100 µL of 3, 5 dinitro salicylic acid reagent (DNSA) and then heated in a boiling water bath for 10 min [40]. Next, the enzymatic activities were estimated spectrophotometrically at λ540 by measuring reducing sugar productions from the substrates using glucose and xylose as standards. The enzyme activities were determined in international units (IU) as described below in the section, “Lignocellulase production by potential isolates”. Based on the screening results, the highest activity-showing isolates were selected, preserved, and evaluated for further studies. 

#### 2.2.7. Growth Profile of the Potential Bacteria in BSM Media Containing Avicel or Xylan

Since the primary isolation and screening of the bacteria were carried out on Avicel and xylan as sole sources of carbon, the growth pattern of the selected isolates was performed by using the same substrates. To determine the growth pattern of the selected potential strains, the freshly prepared bacterial cultures (OD600 ≥ 0.6) were inoculated into the BSM media containing either xylan or Avicel (0.5% *w*/*v*) as carbon sources. The inoculated culture media were agitated in a shaking incubator at 180 rpm and 37 °C until the growth pattern showed a declining phase for each of the bacterium. The culture flasks were monitored for changes in the turbidity as well as optical density at λ600 in a spectrophotometer (Biowave Spectrophotometer, Biochrom Ltd., London, UK) every 12 h. 

#### 2.2.8. Rice Straw and Corn Stover Degradation

The disruption of the corn stover and rice straw by potential bacteria were analyzed as described previously with few modifications [39]. A quantity of 1 mL of the overnight grown inoculum was transferred to a 250 mL Erlenmeyer flask containing 100 mL of BSM media with 2.5% (*w*/*v*) of the respective substrate (rice straw or corn stover). The inoculated culture was then incubated in a rotary shaker at 37 °C and 180 rpm for 7 days. The culture was regularly checked for the change in turbidity of the media and degradation of the substrates. After incubation, the dry weight of the residual lignocellulose was measured, using blank (medium containing carbon sources but no bacterial treatment) as a control. Subsequently the blank readings were subtracted from the test reactions to achieve the weight loss of the substrates. The degradation percent (%) of the substrates were calculated by using the following formula:

$$Degradation\left(\%\right)=\frac{Initial\;wt.of\;substrate-wt.\;of\;substrate\;after\;treatment}{Initial\;wt.of\;substrate}\times 100.$$

#### 2.2.9. Lignocellulase Production by Potential Isolates

The cellulose and hemicellulose degradation capacity of the potential isolates selected on the basis of primary screening were determined through the activities of endoglucanase, exoglucanase, and xylanase. The activities were checked on agricultural waste products, such as rice straw and corn stover, for 7 days. For this, the potential bacteria were inoculated into freshly prepared BSM media containing 2.5% of rice straw or corn stover as the only source of carbon. The bacterial cultures were incubated at 180 rpm and 37 °C in a rotary shaker for a period of 7 days. To optimize the maximum period of incubation and monitor how enzyme activities vary during incubation, small aliquots (≤2 mL) of samples were collected every 24 h. The aliquots were centrifuged at 10,000 rpm for 10 min, and the supernatants obtained were treated as crude enzyme extracts. Endoglucanase activity was determined according to the method described by Nitisinprasert and Temmes [41] with few modifications. The reaction mixture contained 20 μL of enzyme extract and 80 μL of 1% CMC in PBS buffer (pH, 6.0). For endoglucanase activity, the reaction mixture was incubated at 50 °C for 30 min. Exoglucanase (avicellase) activity was assayed by incubating the reaction mixture (20 μL of enzyme extract with 80 μL of 1% Avicel in PBS buffer (pH, 6.0)) at 40 °C for 60 min. During this assay, extensive care was taken to avoid the mixing of the reaction products with Avicel through centrifugation. In these tests, the reactions were terminated by adding 100 μL of DNSA reagent [40] followed by boiling in a water bath for 10 min and then measuring the reducing sugars spectrophotometrically at λ540. The xylanase activity was also determined according to Nitisinprasert and Temmes [41] by incubating a reaction mixture containing 20 µL of enzyme solution appropriately diluted in a PBS buffer (pH, 6.0) with 80 µL of the aqueous suspension of 1% xylan at 50 °C for 30 min. To stop the reaction, 100 μL of DNSA reagent was added to the reactants and then heated for 10 min in a boiling water bath. The amount of reducing sugar released was determined by using D-xylose as standard, while for endo- and exo-glucanase activities, glucose was used as the standard. The enzyme activities were determined in international units (IU) where 1 unit of activity is defined as the amount of enzyme required to liberate 1 µmol of reducing sugar equivalent per min under the standard assay conditions. However, to minimize the assay interference, sample blanks that contained the respective reactants were run for each test but the step of incubation at desired temperatures was omitted. Subsequently, the blank readings were subtracted from the test reactions to achieve the appropriate values. 

#### 2.2.10. Statistical Analysis

The data were subjected to statistical analysis using Microsoft office suite 2016; graphs were plotted in Origin Lab. software version 8.1. The results are reported as means ± standard deviations of three or more independent replicates. For some parameters, the principal component analysis was carried out in the R studio program represented with biplots of the first two principal components [42].

## 3. Results

### 3.1. Identification of the Termites

The COII gene sequencing and phylogenetic reconstruction of the collected termites revealed that the termites belonged to three different species (Figure S1). Group 1 termites belonged to *A. pakistanicus* while group 2 termites were affiliated with *O. longignathus*. Unlike other termites, group 3 termites shared a phylogeny with the *Macrotermes* species. The COII gene sequences of the collected and sequenced termites can be obtained from the Genbank database under accession numbers MZ713168, MZ713177, and OQ716402 for *A. pakistanicus, O. longignathus,* and *Macrotermes* sp., respectively.

### 3.2. Isolation of Bacteria from the Guts of the Termites

To determine the diversity of the culturable lignocellulolytic bacteria within the termites, the gut homogenates were spread on the BSM plates and incubated for enzyme productions. Initially, a total of 80 isolates were purified from all three termite species under consideration. Among these, 14 isolates showed similar morphology and colony characteristics such as color, shape, and size; therefore, only the unique colonies with the least similar features were selected and characterized for Avicel and xylan degradation. A total of 66 unique isolates were found which grow on Avicel and xylan plates. Among the termites, 23, 18, and 25 bacteria were isolated from *A. pakistanicus*, *Macrotermes* sp., and *O. longignathus*, respectively (Table 1). The termite *O. longignathus* was found to contain the maximum number of isolates, while only 18 isolates were represented by *Macrotermes* sp. The majority of the bacteria were isolated from BSM media containing 1% xylan, indicating the easy access and degradation of the xylan polymer. Out of the 66 isolates, the majority (72.7%) of the bacteria were obtained from xylan plates; only 18 isolates (27.3%) showed growth on the microcrystalline cellulose also known as Avicel. After isolation, the bacteria were coded, characterized for phylogenetic analyses, and identified by using a molecular method based on 16S rRNA gene amplification and sequencing. 

### 3.3. Identification and Phylogenetic Analysis of the Bacterial Isolates 

**Figure 1:**
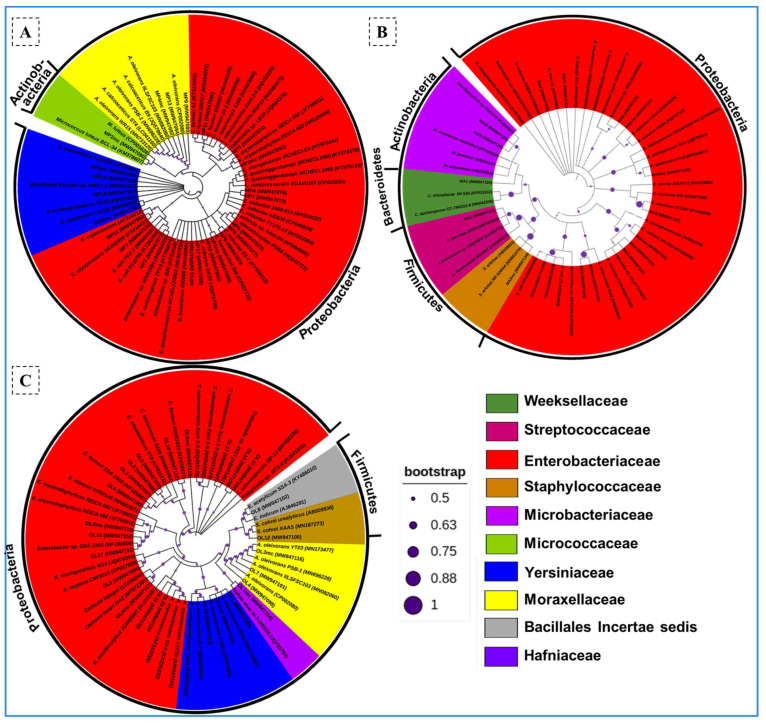
Phylogenetic affiliation of the bacteria isolated from *A. pakistanicus* (**A**), *Macrotermes* sp. (**B**)*,* and *O. longignathus* (**C**). The filled circles indicate the fraction of bootstrap values based on 500 replicates. The phylogenetic trees were reconstructed by the neighbor-joining method in MEGA X software, showing the relationship of the identified bacteria with closely related species retrieved from the NCBI database. The accession number of each isolate is given in parentheses beside the isolate code.

The phylogenetic reconstruction of the isolates revealed bacteria belonging to four bacterial phyla, namely, Proteobacteria, Firmicutes, Actinobacteria, and Bacteroidetes (Figure 1). The majority of the strains belonged to the phylum Proteobacteria represented by 87.8% of the total isolates. The Actinobacteria and Firmicutes were represented by 4.5% and 6%, respectively. However, only a single strain affiliated with the phylum Bacteroidetes was obtained from the gut of *Macrotermes* sp. Among the termite species tested in the present study, *Macrotermes* sp. was found to contain the maximum number of bacterial isolates that belonged to four phyla. The members of the Actinobacteria and Firmicutes were not obtained from the gut systems of *O. longignathus* and *A. pakistanicus*, respectively. At the family level, bacteria belonging to ten different families were isolated from all three termites under consideration. About 68.1% of the isolates were affiliated with Enterobacteriaceae followed by Yersiniaceae (10.6%) and Moraxellaceae (9%). The bacteria isolated from *A. pakistanicus* and *Macrotermes* sp. were affiliated with four families each. The members of Weeksellaceae, Microbacteriaceae, and Streptococcaceae were unique to the gut of *Macrotermes* sp. Similarly, the members of the Bacillales and Micrococcaceae were unique to the gut system of *O. longignathus* and *A. pakistanicus*, respectively. The members of the Moraxellaceae and Yersiniaceae were common among the *A. pakistanicus* and *O. longignathus*. Among the genera observed, *Enterobacter* was the most dominant genus occupying about 22.7 % of the total bacteria followed by *Citrobacter* (13.6%), *Serratia* (10.6%), and *Acinetobacter* (9.09%). 

### 3.4. Termite-Specific Isolates 

**Figure 2:**
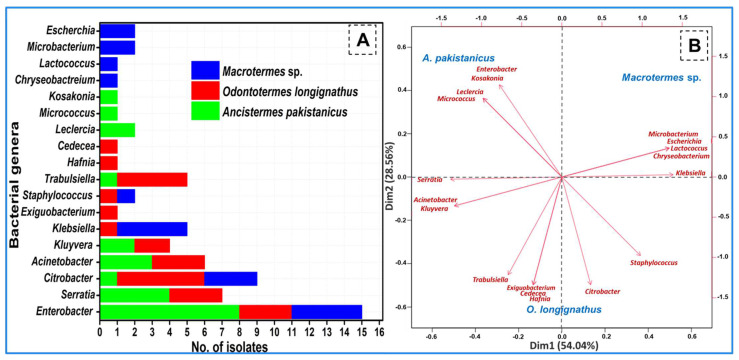
Genera-based distribution of Avicel- and xylan-hydrolyzing bacteria isolated from the gut systems of different species of fungus-growing termites. Genus-wise relative abundance of the o-served bacteria (**A**) and PCA (**B**) indicates the dominance of particular genus in specific termites.

**Figure 3:**
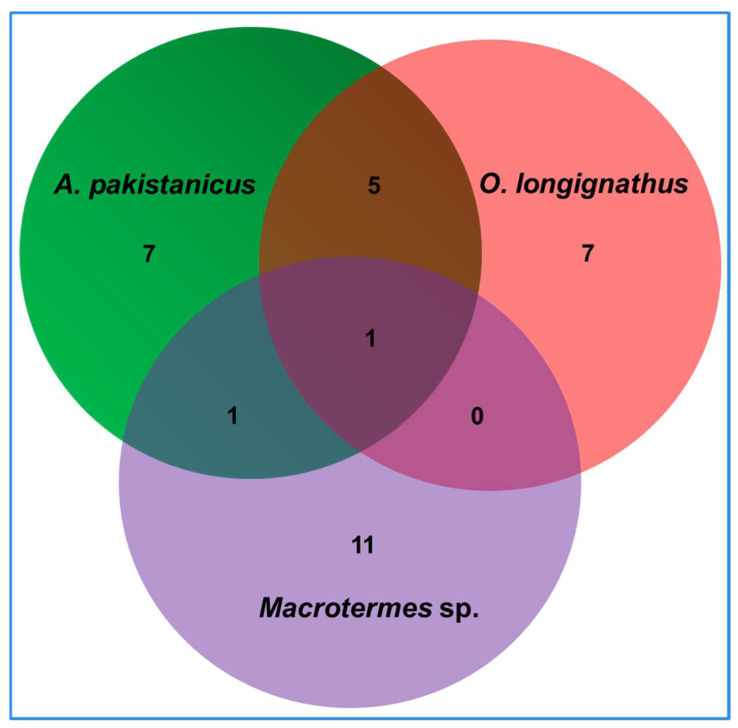
The Venn plot demonstrates the number of unique and shared bacterial species between the fungus-growing termites under consideration. The bacteria were identified after their capability to grow on Avicel and xylan substrates.

**Figure 4:**
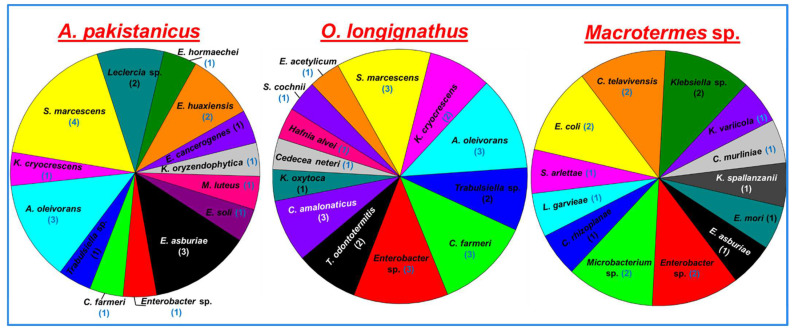
Species-wise diversity and richness of Avicel- and xylan-hydrolyzing bacteria isolated from different fungus-growing termites. The numbers given in brackets after bacteria indicate the number of isolates of a particular species.

The isolated bacteria belonged to 18 different genera of the bacteria (Figure 2). The termite *O. longignathus* harbored the maximum number of bacterial genera (11 genera). Only members of the eight genera of bacteria were observed in the termite *Macrotermes* sp. while *A. pakistanicus* was found to contain bacterial species from nine different genera. Many of the genera were found to be termite-specific as revealed by the PCA analysis which signified uniqueness of the bacterial community. The members of the genera *Chryseobacterium*, *Lactococcus*, *Microbacterium,* and *Escherichia* were unique to the *Macrotermes* sp., while the genera of *Exiguobacterium*, *Hafnia,* and *Cedecea* were observed in the gut of *O. longignathus* only. The genera *Leclercia*, *Micrococcus,* and *Kosakonia * were unique to the gut system of the *A. pakistanicus*. The members of *Citrobacter* and *Enterobacter* were common among the termites. The genera *Acinetobacter, Trabulsiella,* and *Kluyvera* were common among *O. longignathus* and *A. pakistanicus*. Similarly, the members of the genus, *Staphylococcus* were found to be common among *Macrotermes* sp. and *O. longignathus*. A total of 32 species of bacteria were observed in the present study from all three termite species (Figure 3). In terms of the number of species, the highest number (14 isolates accounted for 43.75%) of bacteria were observed in the gut system of the *A. pakistanicus*. The termites *Macrotermes* sp. and *O. longignathus* were represented by 13 species each. Although more species were observed in the gut of *A. pakistanicus*, its Shannon equitability index (H index) was low, i.e., 0.94, when compared to *O. longignathus* (H index = 0.96). Despite only 18 isolates being observed in *Macrotermes* sp., it was represented by 13 different species, thereby depicting the highest Shannon diversity index with 0.98. *Ancistrotermes pakistanicus* was represented by unique species of *Kosakonia oryzendophytica*, *Micrococcus luteus*, *Enterobacter hormaechei*, *E. huaxiensis*, and *E. soli* (Figure 4). The species of *K. spallanzanii*, *Escherichia coli*, *Staphylococcus arlettae*, and *C. murliniae* were unique to the *Macrotermes* sp. 

### 3.5. Bacterial Screening for Cellulase and Xylanase Production 

After identification, the isolated bacteria were tested for the degradation of Avicel and xylan in BSM media. The primary screening revealed that 40 bacterial strains (66.6% of the total isolates) showed discernible activities indicating the production of either avicellase or xylanase (Table 2). Upon primary screening, the average avicellase activity of the bacteria ranged between 0.046 IU/mL extract and 0.35 IU/mL extract over the tested incubation period. The highest avicellase activity was displayed by *Klebsiella spallanzanii* MA4MC with a value of 0.35 IU/mL extract after 3 days of incubation on BSM media containing Avicel as a carbon source. After *K. spallanzanii* MA4MC, the higher avicellase activities were shown by *Enterobacter chengduensis* MA11 (0.34 ± 0.004 IU/mL extract) and *K. cryocrescens* OL10 with a value of 0.18 IU/mL extract. 

**Figure 5:**
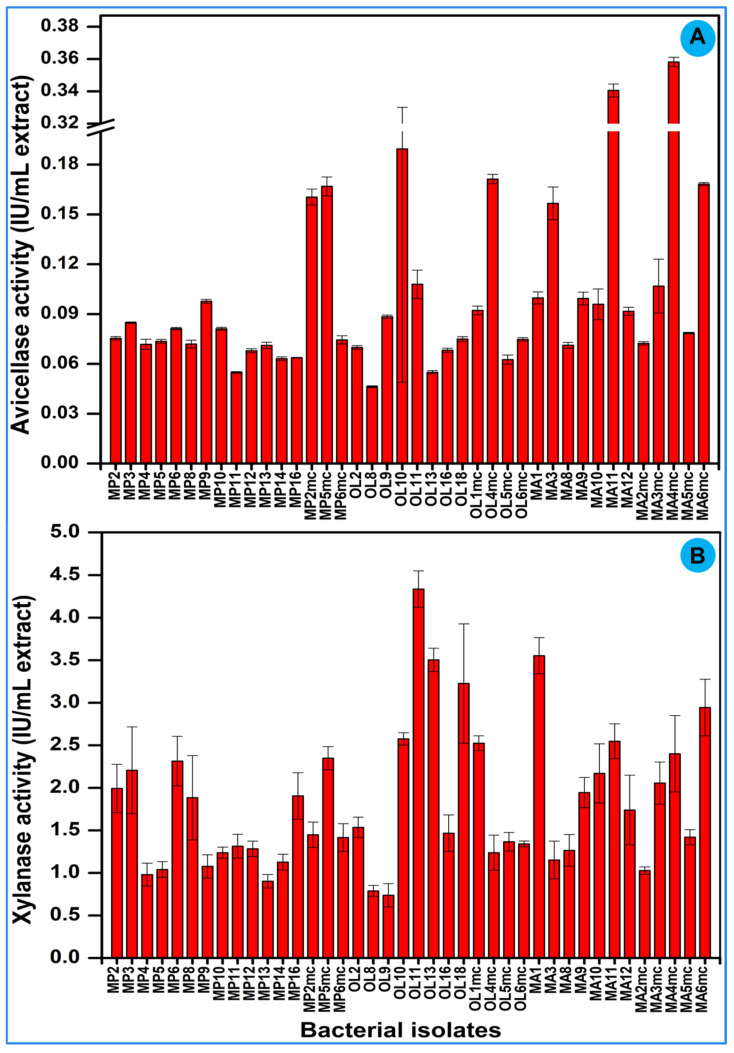
Screening assay for the isolation of lignocellulose-degrading bacteria. The avicellase (**A**) and xylanase (**B**) activities were detected on Avicel and xylan, respectively. The enzyme extracts were harvested after 72 h of culturing.

In contrast to avicellase activities, the xylanase activities of the isolated bacteria were higher showing a 12-fold increase (Figure 5). The higher activities on xylan could be caused due to the less crystalline nature and the pentose rings present in the compound. The xylanase activity of the bacteria ranged between 0.73 IU/mL extract and 4.33 IU/mL extract; the highest was exhibited by *K. cryocrescens* OL11. The lowest xylanase activity was displayed by the bacterium *C. amalonaticus* OL9, with a value of 0.73 IU/mL extract. After *K. cryocrescens* OL11, the maximum xylanase activities were shown by *Chryseobacterium rhizoplanae* MA1 and *Trabulsiella odontotermitis* OL13, displaying 3.55 ±0.2 IU/mL extract and 3.50 ± 0.1 IU/mL extract, respectively. Based on the potential avicellase and xylanase activities, six isolates were selected and further characterized for substrate degradation and enzyme production. 

### 3.6. Growth of Potential Bacteria in the BSM Media Containing Avicel or Xylan 

**Figure 6:**
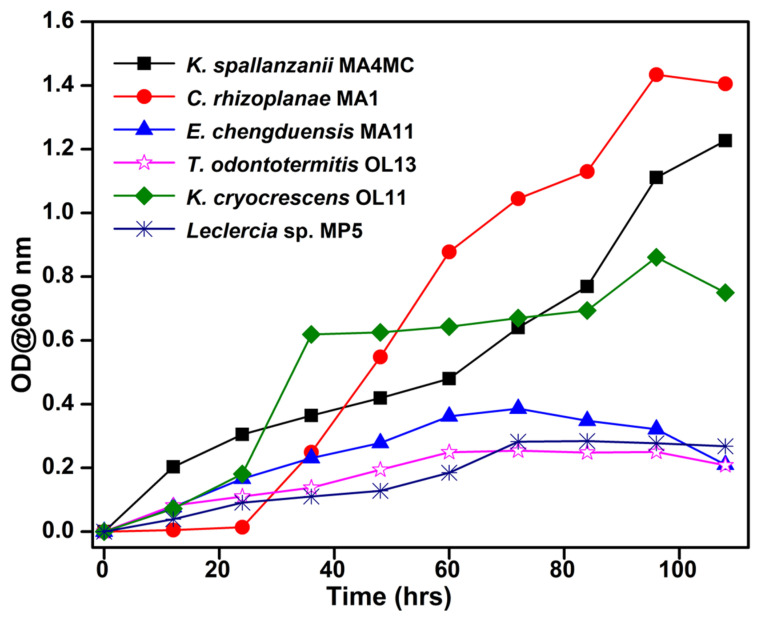
Growth profile of the potential cellulose and hemicellulose degrading bacteria on the BSM media containing 0.5% (*w*/*v*) of Avicel or xylan as carbon sources.

To determine the growth profile of the potential strains, the growth curve analyses were performed by recording the absorbance (OD) at λ_600_ as a measure of the increase in cell number. The absorbance of the bacterial strains in BSM using xylan as a carbon source revealed that the fastest cell proliferation was shown by *C. rhizoplanae* MA1, which continued to grow until 96 h of incubation at 37 °C (Figure 6). The least growth was observed with *T. odontotermitis* OL13 which reached a maximum absorbance of 0.25 after 96 h of incubation. The strain *E. chengduensis* MA11 reached a plateau stage only after 72 h showing a 0.38 absorbance value at OD_600_ nm. However, the bacterium *K. spallanzanii* strain MA4MC showed continuous proliferation of cell numbers throughout the incubation period on Avicel. The growth pattern of other strains followed a similar trend. The slow growth pattern of most strains indicated adaptation to the culture conditions and an inducible nature that can be exploited for fermentation processes.

### 3.7. Rice Straw and Corn Stover Degradation

**Figure 7:**
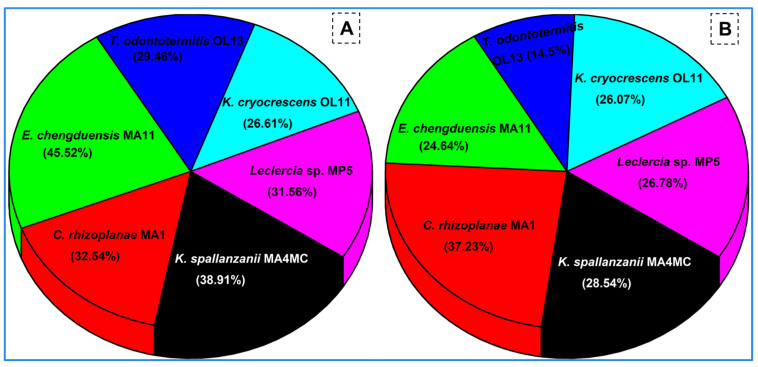
Substrate degradation capability of the potential isolates on rice straw (**A**), and corn stover (**B**) used as sole sources of carbon.

The substrate degradation potential of the selected bacteria was tested on agricultural wastes to determine their capability for lignocellulose bioconversion. Among the six potential bacteria, the highest substrate degradation ratio of 45.52% was demonstrated by *E. chengduensis* MA11 on rice straw (Figure 7). However, the highest corn stover degradation of 37.3% was achieved with *C. rhizoplanae* MA1. The overall substrate degradation was accomplished better on rice straw as compared to corn stover by all the tested strains. The lowest corn stover degradation was shown by *T. odontotermitis* OL13 which could degrade only 14.3% of the substrate. 

### 3.8. Lignocellulase Production by Potential Isolates

Lignocellulose is a recalcitrant substrate due to its complex nature. Cellulose and hemicellulose are the principal components of lignocellulose. They are degraded by a complex of enzymes called cellulases and hemicellulases. The main enzymes of the cellulase complex are endoglucanase and exoglucanase. Endoglucanase, also known as CMCase, randomly attacks the β-1, 4 glycosidic linkages of the cellulose molecule breaking it into short stretches which are then acted upon by exoglucanases that release the terminal disaccharide in the form of cellobiose. Similarly, the hemicelluloses are hydrolyzed by the xylanase complex. Therefore, determining the cellulolytic and xylanolytic potential of the isolated bacteria becomes imperative for their potential biotechnological applications. 

#### 3.8.1. Endoglucanase Activity

When tested on rice straw, the highest endoglucanase activity was shown by *K. cryocrescens* OL11 after 24 h of incubation with a value of 0.47 IU/mL extract. The highest endoglucanase activity on corn stover was displayed by *C. rhizoplanae* MA1. Among the tested isolates, the lowest endoglucanase activities were shown by *Leclercia* sp. MP5 (0.05 IU/mL extract) and *K. cryocrescens* OL11 (0.06 IU/mL extract) on corn stover and rice straw, respectively, after 4 days of incubation (Table 3 and Table 4). Although the pattern of endoglucanase production was the same on both substrates, the activities on rice straw were comparatively higher. 

#### 3.8.2. Exoglucanase Activity

Exoglucanase activity, which is a measure of the degradation of crystalline cellulose, was found to be at its highest on rice straw after 2 days of incubation. Among the tested carbon sources, the highest activity of 0.64 IU/mL extract was shown by *C. rhizoplanae* MA1 (Table 3). The highest exoglucanase activity on corn stover was achieved on the first day by *K. spallanzanii* MA4MC with a value of 0.46 IU/mL extract (Table 4). Similar to the endoglucanase activity, the lowest exoglucanase activity of 0.14 IU/mL extract on rice straw was shown by *K. cryocrescens* OL11 after 3 days of incubation. The lowest activity (0.13 IU/mL extract) on corn stover was detected after 6 days of incubation by *Leclercia* sp. MP5. Overall, the exoglucanase activities on both tested substrates were higher than the endoglucanase activities. This increase in exoglucanase activity could be due to the initial culturing and isolation of the bacteria on Avicel as the sole source of carbon. 

#### 3.8.3. Xylanase Activity

Xylanase activity, representing the hydrolysis of pentose sugars of hemicellulose content of lignocellulosic biomass, was observed highest on rice straw by *K. spallanzanii* MA4MC with 2.99 IU/mL extract (Table 3). The highest xylanase activity on corn stover was observed after 2 days of incubation with the bacterium *T. odontotermitis* OL13 showing a value of 1.80 IU/mL extract, while the lowest activity of 0.26 IU/mL extract was achieved with *K. spallanzanii* MA4MC after 7 days of incubation (Table 4). However, on rice straw, the lowest xylanase activity (0.98 IU/mL extract) was observed on the first day of incubation by *C. rhizoplanae* MA1. A notable feature was the 1.6-fold increase in xylanase activity on rice straw as compared to the highest activity on corn stover. The xylanase activity on rice straw was also many times higher than the endo- and exo-glucanase activities on the tested substrates. 

## 4. Discussion 

Termites are known to digest the lignocellulosic biomass efficiently through the contribution of gut symbionts such as bacteria [43,44]. Despite several studies based on metagenomics that have reported the occurrence of bacterial symbionts within termite guts, only a few studies have focused on evaluating the potential of individual gut symbiotic bacteria in fungus-growing (higher) termites. Particularly, the functions of the culturable bacterial symbionts within the gut of fungus-growing termites *A. pakistanicus* and *O. longignathus* are still unknown. To this end, the present study comparatively demonstrates and compares the diversity of lignocellulolytic bacterial players in the gut systems of three different fungus-growing termite species viz, *A. pakistanicus*, *O. longignathus*, and *Macrotermes* sp. via culturable methods. In addition, the potential contribution of the individual bacteria involved in the lignocellulose degradation, particularly cellulose and hemicellulose hydrolysis, has been revealed to decode the symbiotic mechanisms between the termite and its symbionts. Moreover, to the best of our knowledge, this is the first study to report the symbiotic bacteria functioning towards lignocellulose digestion within the gut of the fungus-growing termite *A. pakistanicus*. In this perspective, we believe that the present study elaborates the profile of the cellulose- and hemicellulose-degrading bacteria of the termites in general and fungus-growing species in particular. 

The molecular phylogeny revealed that termites harbor a tremendous diversity of lignocellulose-digesting bacterial symbionts in their gut systems. The isolated bacteria were affiliated with four major phyla: Proteobacteria, Firmicutes, Actinobacteria, and Bacteroidetes. The culturable cellulolytic members of these phyla have been frequently reported from other termite species [45,46,47]. Previously, we have also isolated the members of similar phyla from the gut regions of the wood-feeding termite *Coptotermes formosanus* [43]. The dominance of Proteobacteria in the gut systems is related to its sensitivity to environmental factors such as diet, working as a frontline responder to the pathogens ingested through food [48]. Among Proteobacteria, the dominance of Enterobacteriaceae corresponds with the low pH and high partial pressure of the gut systems of termites and cockroaches [49]. The absence of the Spirochaetes and Clostridiales is surprising because these bacteria are known to be dominant groups within termite guts [50,51]. The noticeable absence of these bacteria could be due to the specificity of culture media together with carbon sources and the aerobic conditions used in the study [52,53], as a majority of the *Clostridium* species known from animal guts are strict anaerobes [54]. 

The PCA analysis demonstrated a unique distribution of the isolated bacteria in the gut systems of termites. The PCA analysis revealed a dominance of the bacterial genera *Enterobacter*, *Kosakonia*, *Leclercia,* and *Micrococcus* in the gut system of *A. pakistanicus*, while *Staphylococcus* and *Citrobacter* showed a positive correlation towards the gut of *O. longignathus*. In contrast, the members of *Microbacterium*, *Lactococcus,* and *Chryseobacterium* were more dominant in *Macrotermes* species. Many of the bacteria were constant among the tested termites. The genera-based PCA analysis further grouped the members of *Acinetobacter*, *Trabulsiella*, *Serratia*, and *Kluyvera* as common among the *O. longignathus* and *A. pakistanicus*. The isolation of the *Leclercia* sp., *Acinetobacter*, and *Staphylococcus* have been reported in the fungus-growing termites *Odontotermes formosanus*, as well as the wood-feeding species *Mironasutitermes shangchengensis* and *Microcerotermes diversus* [55,56]. The occurrence of *Citrobacter* spp. in all the termites under consideration is in line with other studies [57] which detected them in the hindgut of lower wood-feeding termites. The ubiquitous presence of these bacteria in termites comprises a constant fraction of gut microbiota that can be regarded as core bacteria. These core bacteria are vital for host fitness due to their involvement in carbohydrate digestion, nitrogen metabolism, and fermentation besides pheromone production and immunity [58]. 

The gut symbiotic bacteria in termites are recognized as active players that contribute to the host’s metabolism [59]. In fungus-growing termites, the lignocellulose partially digested by symbiotic *Termitomyces* sp. is completely metabolized through the contributions from gut bacteria and endogenous enzymes [18,60]. To achieve this, the partially hydrolyzed wood fragments along with fungal nodules are ingested by the host termites [18] and exposed to endogenous proteins and salivary endoglucanases [61] for further breakdown of amorphous cellulose. The remaining diet/lignocellulose containing a high amount of crystalline cellulose is finally hydrolyzed by bacterial enzymes housed in the gut system [62,63]. *Enterobacter* and *Klebsiella* are common in many lignocellulose-feeding insects [64], where they significantly contribute to lignocellulose digestion [65,66]. The members of the *Enterobacter* genus have been frequently isolated from termite guts where they act on four different compounds: cellulose, hemicellulose, lignin, and other aromatic compounds [57,67]. Based on the literature, these bacteria contribute to the second stage of lignocellulose degradation [68]. Recently, two cellulose hydrolyzing bacterial strains (M21WG and Z6WG) of the genus *Klebsiella* have been isolated from the gut of mound-building termites [69]. These bacteria were found to utilize up to 1.5% of CMC substrate at 37 °C and pH 7 [69] suggesting their symbiotic role with termites for digestion. The CMC degradation activity of the *Leclercia* sp. MP5 is in congruence with the observations of previous studies that detected these bacteria in wasps. The CMC hydrolyzing *Leclercia* species have been previously isolated from an invasive wood-feeding wasp *Sirex noctilio* [70]. Similarly, the members of *Acinetobacter* and *Klebsiella* are known to depolymerize cellulose within the insect gut [71]. The cellulase-encoding gene of the *Acinetobacter*, isolated from the gut of *Gryllotalpa Africana*, has been successfully cloned by Banerjee et al. [72]. The expressed and purified enzyme of the *Acinetobacter* has been reported to degrade sawdust, which is also fed upon by the termites, thereby signifying their support to the host. The isolation of *Staphylococcus* species on Avicel and xylan is in congruence with previous studies [56,69]. These bacteria are known to contribute cellulases and hemicellulases of the glycoside hydrolase families GHF10 and GHF11, [73,74] to the host for the catabolism of ingested carbohydrates. 

The cellulase production of the *K. cryocrescens* was consistent with previous findings [75] where the authors isolated these bacteria from the gut of the red panda (*Ailurus fulgens*) and the beetle *Tribolium castaneum* [64]. The symbiotic functions of *T.* *odontotermitis* within the paunch compartment of fungus-growing termites are also suggested by previous studies [76,77]. To date a limited number of the *Trabulsiella* species have been discovered, the majority of them being from the gut system of animals including termites such as *O. formosanus* [77] and *C. formosanus* [78]. Among them, *T. odontotermitis* is specific to termites only. Recently, two cellulase-encoding genes (egl-FZYE and cel-FZYE) that represent endoglucanase and β-glucosidase, respectively, have been cloned from the gut bacterium *T. odontotermitis* [79]. The expressed enzymes have been reported to effectively degrade agricultural wastes such as corn stover, pine sawdust, and sorghum stover [79]; this agrees with our observations. The cellulose-degrading *Chryseobacterium* species have been isolated from the gut system of cockroaches and termites [80]. The *Chryseobacterium* strain FR2 has been applauded for its fermentation capacity within the cockroach gut, releasing short-chain fatty acids that are directly absorbed by the host with hemolymph. Although many species of the genus *Chryseobacterium* have been detected in termites [81], to date none of the studies have observed *C. rhizoplane* within termite guts. In this perspective, the present study elaborates the description of the cellulase encoding bacterial species of the termites which might be helpful to understand the biology of the termite-bacteria symbiosis.

Out of the total 66 isolates, forty bacteria showed the production of cellulase and xylanases. This signifies the synergism of these isolates during saccharification and metabolism of the lignocellulose within the termite gut. These results are supported by the fact that many of these strains could secrete multienzyme complexes, including endoglucanases, exoglucanases, and xylanases. Considering that endoglucanase cleaves high-molecular-weight cellulose into low-molecular-weight cellulose chains, most of the strains showed all three enzyme activities under consideration [82]. The endogenous secretion of endoglucanases by the salivary glands and the hindgut of termites is a well-known phenomenon [83,84,85]. The endoglucanase activities of the potential bacteria were comparatively lower than their exoglucanase and xylanase activities, which indicate that endoglucanase enzymes are usually contributed by the host [6]; thus, these bacteria have, over time, lost their ability to express endoglucanase due to long-term symbiosis [81]. In other words, the lower endoglucanase activities of bacteria imply their coevolution within the gut systems to synergize the efficient digestion of lignocellulose with potential contributions from hosts that resulted in the diminution of some enzyme activities.

The comparison of the cellulolytic activities of potential strains with previous studies is challenging due to several reasons (e.g., culture media, fermentation conditions, and substrates) related to the extracellular production of required enzymes. However, here we have identified a collection of newly isolated bacterial symbionts from different termites. These are *C. rhizoplanae* MA1, *C. murliniae* MA8, *K. spallanzanii* MA4MC, *C. neteri* OL4MC, *L. decarboxylata* MP6, *E. oryzendophyticus* OL16, and *H. alvei* OL1MC. Although the genera-level resolution of these bacteria has been defined in termites previously, here we describe their species-level identification as well as their functions towards the host metabolism. The observed results are encouraging as the potential bacteria isolated, evaluated, and characterized in this study might represent a unique biological resource for the conversion of lignocelluloses into value-added commercial products such as reducing sugars and biofuels. In conclusion, many new multifunctional lignocellulase-producing bacteria have been isolated that have a synergistic effect on cellulose and hemicellulose degradation. This process is fundamental for industrially relevant biotechnological applications because it provides a microbial source of novel multifunctional enzymes that can improve the efficiency of the hydrolysis of lignocellulosic biomass into fermentable sugars. Taken together, the findings indicate the tremendous potential of termite gut bacteria that can be exploited for several biotechnological applications after a thorough understanding of the involved mechanisms.

## 5. Conclusions 

The culture-dependent isolation and diversity index of the bacteria revealed that fungus-growing termites may harbor a rich diversity of prokaryotic symbionts involved in lignocellulose degradation. To the best of our knowledge and based on the available literature, the present study reveals a unique microbial diversity in different termites with some distinct bacterial species that are confined to termite guts. Furthermore, based on the evaluation and characterization of some potential isolates, the symbiosis of the host with gut bacteria for lignocellulose bioconversion has been identified. The overall results indicate that *O. longignathus*, *A. pakistanicus*, and *Macrotermes* termites may serve as novel reservoirs of hydrolytic enzymes for application in the conversion of lignocellulose into biofuels. A limited number of bacterial species were detected in the present study when compared with previous reports that have used metagenomics. This could be due to the applied culture conditions using a specific culture medium and carbon sources, such as xylan and Avicel, that may have limited the growth of anaerobic and unculturable bacterial species [86]. Therefore, a good picture of the bacterial diversity residing in the gut system of these termites would likely be deciphered by integrating next-generation sequencing tools. Correspondingly, the future perspectives of this study will be metagenomic and metatranscriptomic analyses of the bacterial symbionts and their functions within the termite gut systems to decode the bio-catalytic networks involved in biomass conversion. Moreover, the potential strains selected here will be further characterized, along with cloning and expressing their cellulase genes into non-cellulolytic strains such as *E. coli* for large-scale enzyme production.

## Figures and Tables

**Table 1:** Profile of the culturable bacteria within the termite gut systems along with their isolation sources and substrates showing lignocellulase activities.

Termite	Number of Isolates (%)	Number of Isolates Obtained from Xylan (%)	Number of Isolates Obtained from Avicel (%)	Activity Showing Isolates (%)
*Ancistrotermes pakistanicus*	23 (34.8)	17 (74)	6 (26)	16 (69.5)
*Odontotermes longignathus*	25 (37.8)	19 (76)	6 (24)	12 (48)
*Macrotermes* sp.	18 (27.2)	12 (66.6)	6 (33.3)	12 (66.6)
Total	66	48 (72.7)	18 (27.3)	40 (60)

**Table 2:** A detailed account of the identification, source, and activity of the lignocellulolytic isolates along with their percent similarity of the corresponding 16S rDNA genes to closely related NCBI bacterial species determined by BLASTn search.

Sr. No.	Isolate Code	Termite Species	Accession Number	NCBI Likely Species (Accession Number)	Similarity (%)	Query Cover (%)	Carbon Source Used for Isolation	Activity (IU/mL Extract)
1	MA1	*Macrotermes* sp.	MW947120	*Chryseobacterium rhizoplanae* (KP033261)	97.11	100	Xylan	3.55
2	MA3		MW947122	*Klebsiella* sp. (KT716257)	100	100		1.15
3	MA8		MW947127	*Citrobacter murliniae* (KY436219)	100	100		1.26
4	MA9		MW947128	*Microbacterium* sp. (KJ200387)	99.95	96		1.94
5	MA10		MW947129		99.93	99		2.17
6	MA11		MW947130	*Enterobacter chengduensis* WCHECl-C4	99.85	100		2.54
7	MA12		MW947131	*Escherichia coli* C7 (HG941663)	99.93	100		1.73
8	MA2MC		MW947133	*Escherichia coli* W5 (MN086364)	100	100	Avicel	0.07
9	MA3MC		MW947134	*Staphylococcus arlettae* (MN851074)	100	100		0.10
10	MA4MC		MW947135	*K. spallanzanii* Ko3 (MN104674)	99.85	100		0.35
11	MA5MC		MW947136	*Citrobacter telavivensis* 6105 (MN603664)	98.93	100		0.07
12	MA6MC		MW947137	*Enterobacter asburiae* (MG516126)	99.50	98		0.16
13	OL2	*Odontotermes longignathus*	MW947096	*Klebsiella oxytoca* (CP017928)	99.64	100		1.53
14	OL8		MW947102	*Exiguobacterium acetylicum* (KY486010)	100	100		0.78
15	OL9		MW947103	*Citrobacter amalonaticus* (CP011132)	99.50	100		0.73
16	OL10		MW947104	*Kluyvera cryocrescens* (AF310218)	99.78	100		2.57
17	OL11		MW947105		99.86	100		4.33
18	OL13		MW947107	*Trabulsiella odontotermitis* Of17 (AB673461)		100		3.50
19	OL16		MW947110	*Enterobacter oryzendophyticus* REICA_082 (JF795011)	98.86	100		1.46
20	OL18		MW947112	*Citrobacter farmeri* (KF475827)	99.72	100		3.22
21	OL1MC		MW947114	*Hafnia alvei* (JQ795764)	100	100	Avicel	0.09
22	OL4MC		MW947117	*Cedecea neteri* (MT072184)	100	100		0.17
23	OL5MC		MW947118	*Enterobacter oryzendophyticus* REICA_082 (JF795011)	98.72	100		0.06
24	OL6MC		MW947119	*Trabulsiella odontotermitis* strain Eant 3–9 (DQ453129)	98.83	99		0.07
25	MP2	*Ancistrotermes pakistanicus*	MW947073	*K. cryocrescens* (KX0184413)	99.93	100	Xylan	1.99
26	MP3		MW947074	*Trabulsiella* sp. LB10 (JQ864379)	99.5	99		2.20
27	MP4		MW947075	*Enterobacter asburiae* (AP019632)	99.93	99		0.98
28	MP5		MW947076	*Leclercia* sp. 6AM.I94 (MT084586)	99.93	100		1.04
29	MP6		MW947077	*Leclercia decarboxylata* (HQ242721)	99.93	99		2.31
30	MP8		MW947079	*Enterobacter asburiae* (KY938112)	99.93	100		1.88
31	MP9		MW947080	*Acinetobacter oleivorans* (CP002080)	100	100		1.07
32	MP10		MW947081	*Enterobacter cancerogenes* (KT766075)	99.93	99		1.23
33	MP11		MW947082	*Citrobacter farmeri* (MH542295)	100	100		1.31
34	MP12		MW947083	*Kluyvera cryocrescens* (KX018413)	99.72	100		1.28
35	MP13		MW947084	*Acinetobacter oleivorans* (CP002080)	100	100		0.90
36	MP14		MW947085	*Serratia marcescens* (CP033623)	99.93	100		1.12
37	MP16		MW947087	*Serratia surfactantfaciens* YD25 (KM093865)	99.78	100		1.90
38	MP2MC		MW947090	*Micrococcus luteus* (KM378607)	100	100	Avicel	0.16
39	MP5MC		MW947093	*Serratia marcescens* (MN368507)	100	100		0.16
40	MP6MC		MW947094	*Serratia marcescens* (CP033623)	100	100		0.07

**Table 3:** Profile of the enzyme activities of potential isolates during the growth on rice straw.

Enzyme Activity (IU/mL Extract)	Incubation (Days)	*K. michiganensis* MA4MC	*C. rhizoplanae* MA1	*E. chengduensis* MA11	*T. odontotermitis* OL13	*K. cryocrescens* OL11	*Leclercia* sp. MP5
Endoglucanase (CMCase)	1	0.25 ± 0.0	0.33 ± 0.0	0.15 ± 0.0	0.37 ± 0.0	0.47 ± 0.0	0.27 ± 0.0
	2	0.10 ± 0.0	0.10 ± 0.0	0.09 ± 0.0	0.08 ± 0.0	0.09 ± 0.0	0.13 ± 0.0
	3	0.09 ± 0.0	0.10 ± 0.0	0.11 ± 0.0	0.09 ± 0.0	0.13 ± 0.0	0.12 ± 0.0
	4	0.06 ± 0.0	0.07 ± 0.0	0.07 ± 0.0	0.07 ± 0.0	0.06 ± 0.0	0.07 ± 0.0
	5	0.09 ± 0.0	0.10 ± 0.0	0.10 ± 0.0	0.09 ± 0.0	0.09 ± 0.0	0.15 ± 0.0
	6	0.07 ± 0.0	0.17 ± 0.0	0.08 ± 0.0	0.07 ± 0.0	0.16 ± 0.0	0.36 ± 0.1
	7	0.09 ± 0.0	0.24 ± 0.0	0.09 ± 0.0	0.17 ± 0.0	0.10 ± 0.0	0.35 ± 0.1
Exoglucanase (avicellase)	1	0.61 ± 0.1	0.52 ± 0.1	0.38 ± 0.0	0.52 ± 0.2	0.62 ± 0.4	0.46 ± 0.0
	2	0.26 ± 0.0	0.64 ± 0.2	0.37 ± 0.0	0.63 ± 0.2	0.45 ± 0.1	0.29 ± 0.0
	3	0.32 ± 0.0	0.16 ± 0.0	0.38 ± 0.1	0.38 ± 0.1	0.14 ± 0.0	0.42 ± 0.1
	4	0.21 ± 0.0	0.18 ± 0.0	0.48 ± 0.4	0.26 ± 0.1	0.30 ± 0.1	0.22 ± 0.0
	5	0.27 ± 0.0	0.27 ± 0.0	0.32 ± 0.0	0.30 ± 0.0	0.25 ± 0.0	0.30 ± 0.0
	6	0.19 ± 0.0	0.20 ± 0.0	0.32 ± 0.0	0.31 ± 0.1	0.20 ± 0.0	0.26 ± 0.0
	7	0.19 ± 0.0	0.28 ± 0.1	0.32 ± 0.0	0.17 ± 0.0	0.24 ± 0.0	0.20 ± 0.0
Xylanase	1	1.73 ± 0.3	0.98 ± 0.0	2.11 ± 0.2	1.75 ± 0.1	0.45 ± 0.0	1.94 ± 0.1
	2	2.12 ± 0.1	1.93 ± 0.2	2.43 ± 0.1	1.77 ± 0.5	1.82 ± 0.3	2.13 ± 0.0
	3	1.53 ± 0.7	1.78 ± 0.3	2.14 ± 0.2	1.61 ± 0.2	1.95 ± 0.1	1.80 ± 0.3
	4	2.99 ± 0.0	2.54 ± 0.1	2.86 ± 0.2	2.37 ± 0.3	2.91 ± 0.2	2.39 ± 0.4
	5	1.59 ± 0.2	1.70 ± 0.9	2.05 ± 0.2	1.28 ± 0.4	1.95 ± 0.3	2.06 ± 0.1
	6	2.94 ± 0.2	2.28 ± 0.1	2.76 ± 0.1	2.62 ± 0.3	2.67 ± 0.0	2.85 ± 0.2
	7	2.18 ± 0.1	1.79 ± 0.2	2.40 ± 0.2	2.15 ± 0.2	2.12 ± 0.2	2.23 ± 0.2

**Table 4:** Profile of the enzyme activities of the potential isolates during the growth on corn stover.

Enzyme Activity (IU/mL Extract)	Incubation (Days)	*K. michiganensis* MA4MC	*C. rhizoplanae* MA1	*E. chengduensis* MA11	*T. odontotermitis* OL13	*K. cryocrescens* OL11	*Leclercia* sp. MP5
Endoglucanase (CMCase)	1	0.15 ± 0.01	0.19 ± 0.01	0.11 ± 0.03	0.10 ± 0.01	0.11 ± 0.01	0.14 ± 0.0
	2	0.10 ± 0.01	0.10 ± 0.01	0.10 ± 0.0	0.09 ± 0.01	0.09 ± 0.0	0.09 ± 0.0
	3	0.10 ± 0.01	0.09 ± 0.0	0.09 ± 0.0	0.08 ± 0.01	0.09 ± 0.0	0.13 ± 0.0
	4	0.07 ± 0.0	0.06 ± 0.01	0.06 ± 0.01	0.06 ± 0.0	0.06 ± 0.0	0.05 ± 0.0
	5	0.09 ± 0.01	0.08 ± 0.02	0.09 ± 0.02	0.07 ± 0.01	0.08 ± 0.0	0.11 ± 0.0
	6	0.08 ± 0.0	0.09 ± 0.02	0.07 ± 0.0	0.08 ± 0.0	0.09 ± 0.0	0.08 ± 0.0
	7	0.13 ± 0.07	0.08 ± 0.01	0.13 ± 0.01	0.08 ± 0.0	0.07 ± 0.0	0.08 ± 0.0
Exoglucanase (avicellase)	1	0.46 ± 0.02	0.32 ± 0.06	0.16 ± 0.01	0.17 ± 0.05	0.20 ± 0.06	0.29 ± 0.1
	2	0.16 ± 0.02	0.33 ± 0.1	0.36 ± 0.05	0.39 ± 0.08	0.22 ± 0.03	0.23 ± 0.07
	3	0.32 ± 0.1	0.15 ± 0.01	0.21 ± 0.08	0.14 ± 0.04	0.25 ± 0.02	0.38 ± 0.2
	4	0.16 ± 0.02	0.16 ± 0.02	0.14 ± 0.02	0.31 ± 0.2	0.19 ± 0.05	0.22 ± 0.1
	5	0.32 ± 0.05	0.20 ± 0.07	0.15 ± 0.04	0.15 ± 0.01	0.16 ± 0.02	0.15 ± 0.02
	6	0.26 ± 0.02	0.20 ± 0.03	0.26 ± 0.04	0.20 ± 0.08	0.14 ± 0.04	0.13 ± 0.06
	7	0.16 ± 0.02	0.17 ± 0.04	0.14 ± 0.03	0.15 ± 0.05	0.16 ± 0.06	0.15 ± 0.06
Xylanase	1	0.74 ± 0.05	0.61 ± 0.06	0.71 ± 0.05	0.91 ± 0.4	0.53 ± 0.2	0.75 ± 0.4
	2	0.91 ± 0.08	0.97 ± 0.2	0.78 ± 0.4	1.80 ± 0.07	1.12 ± 0.2	1.05 ± 0.08
	3	0.48 ± 0.2	0.67 ± 0.2	0.87 ± 0.3	0.77 ± 0.1	0.67 ± 0.2	1.09 ± 0.01
	4	0.44 ± 0.4	1.23 ± 0.2	0.99 ± 0.2	0.95 ± 0.1	0.80 ± 0.1	1.70 ± 0.06
	5	0.53 ± 0.06	0.84 ± 0.04	0.71 ± 0.2	0.82 ± 0.07	0.68 ± 0.2	1.21 ± 0.4
	6	0.55 ± 0.1	0.33 ± 0.1	0.42 ± 0.2	0.69 ± 0.4	0.57 ± 0.2	0.54 ± 0.2
	7	0.26 ± 0.1	0.65 ± 0.1	0.76 ± 0.2	0.92 ± 0.1	0.72 ± 0.1	0.91 ± 0.1

## Data Availability

All the data sets generated for this research are included in the manuscript or supplement files.

## Supplementary Materials

The following supporting information can be downloaded at: https://www.mdpi.com/article/10.3390/insects14040403/s1, Figure S1: The phylogenetic affiliation of the termite species used in the present study. The phylogenetic tree was reconstructed with the neighbor-joining method in Mega 7 based on the cytochrome oxidase II gene sequences of the termites. The termite species used for the isolation of lignocellulolytic gut bacteria are marked in bold.
