# Supplementary material for: Host-Specific Diversity of Culturable Bacteria in the Gut Systems of Fungus-Growing Termites and Their Potential Functions towards Lignocellulose Bioconversion
Source: Insects. 2023 Apr 21;14(4):403. doi: 10.3390/insects14040403 (PMC10146277; doi:10.3390/insects14040403)
Supplement: Supplementary file 1 [file insects-14-00403-s001.zip › insects-2292934-supplementary.pdf]

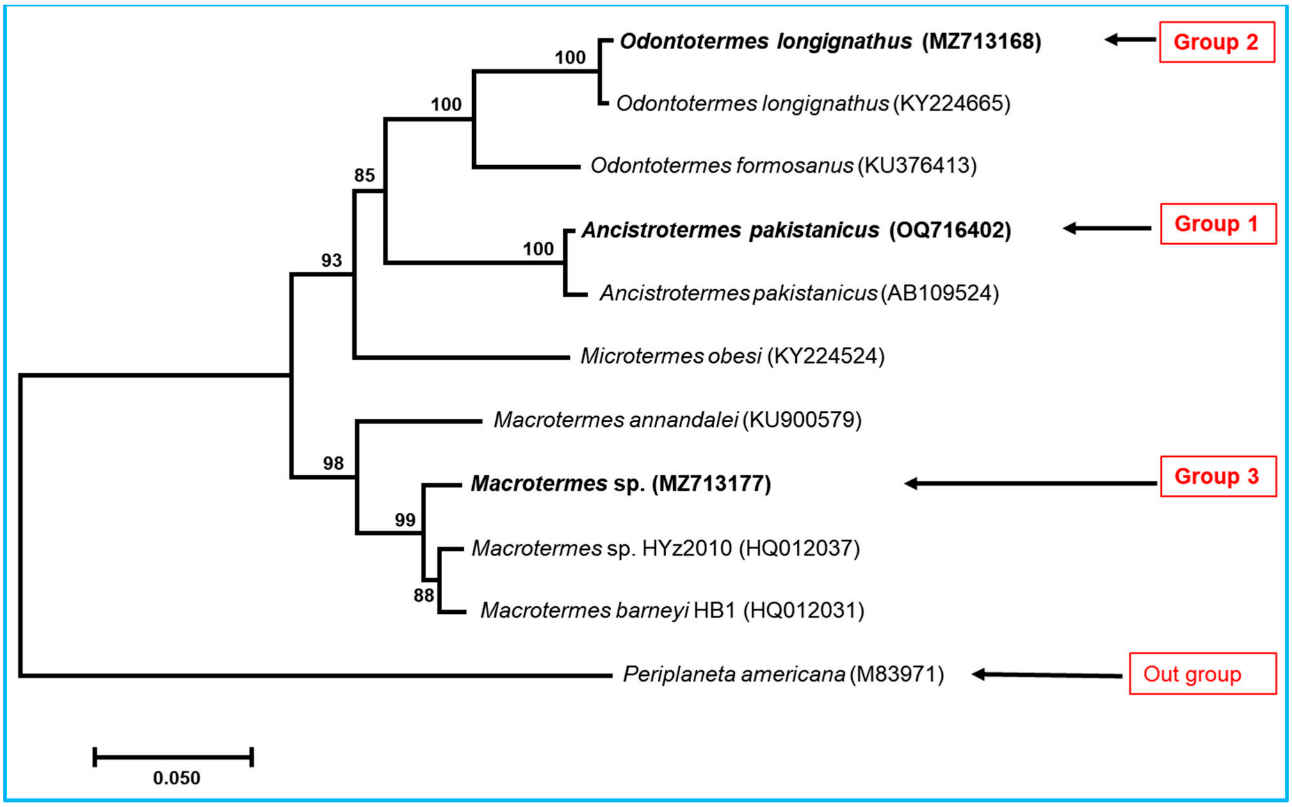

Figure S1. The phylogenetic affiliation of the termite species used in the present study. The phylogenetic tree was reconstructed with the Neighbour-Joining method in Mega 7 based on the cytochrome oxidase II gene sequences of the termites. The termite species used for the isolation of lignocellulolytic gut bacteria are marked in bold.
